# Diagnostic value of circulating miRNA-122 for hepatitis B virus and/or hepatitis C virus-associated chronic viral hepatitis

**DOI:** 10.1042/BSR20190900

**Published:** 2019-09-06

**Authors:** Xinhao Zhou, Shiqiang Fang, Mian Wang, Ali Xiong, Chao Zheng, Jiulong Wang, Changqing Yin

**Affiliations:** 1 Department of Medical Laboratory, The Central Hospital of Wuhan, Tongji Medical College, Huazhong University of Science and Technology, Wuhan 430014, China; 2 School of Laboratory Medicine, Hubei University of Chinese Medicine, Huangjia Lake West Road, Wuhan 430065, China; 3 Department of Oral and Maxillofacial Surgery, The Central Hospital of Wuhan, Tongji Medical College, Huazhong University of Science and Technology, Wuhan 430014, China

**Keywords:** chronic viral hepatitis, diagnosis, meta-analysis, miR-122

## Abstract

**Background:** The liver-specific microRNA-122 (miR-122) has been demonstrated as a powerful and promising biomarker of hepatic diseases. However, the researches on the accuracy of miR122 detection in chronic viral hepatitis have been inconsistent, leading us to conduct this meta-analysis to systematically summarize the diagnostic value of circulating miR-122 in patients with hepatitis B virus (HBV) and/or hepatitis C virus (HCV)-associated chronic viral hepatitis.

**Methods:** A comprehensive literature search (updated to January 30, 2019) in PubMed, Cochrane library, EMBASE, CNKI, Wanfang, and CQVIP databases was performed to identify eligible studies. The sensitivity (SEN), specificity (SPE), positive and negative likelihood ratios (PLR and NLR), diagnostic odds ratio (DOR), and area under the curve (AUC) were pooled to explore the diagnostic performance of circulating miR-122. Subgroup and threshold effect analysis were further carried out to explore the heterogeneity.

**Results:** Overall, 15 studies were finally included in this meta-analysis according to the exclusion and inclusion criteria. The pooled estimates indicated a moderately high diagnostic accuracy for circulating miR-122, with a sensitivity of 0.92 [95% confidence interval (CI), 0.86–0.95], a specificity of 0.84 (95% CI, 0.78–0.89), a PLR of 5.7 (95% CI, 4.7–8.1), a NLR of 0.1 (95% CI, 0.06–0.18), a DOR of 57 (95% CI 25-129), and an AUC of 0.93 (95% CI, 0.91–0.95). The subgroup analysis demonstrated that diagnostic accuracy was better for HCV-associated chronic viral hepatitis patients and non-Chinese compared with other subgroups. In addition, we found that serum might be a more promising matrix for detecting the expression of miR-122 than plasma.

**Conclusions:** Our results demonstrated that circulating miR-122 have a relatively high diagnostic value for chronic viral hepatitis detection, especially in the patients with HCV-associated chronic viral hepatitis. However, further large cohort studies are still required to confirm our findings.

## Introduction

Hepatitis usually refers to inflammation of the liver tissue, which may result from both infectious (e.g. viral and bacterial) and noninfectious causes (e.g. alcohol, certain medications, and toxins). Severe liver disease usually contributes to persistent inflammation and necrosis, of which the two primary adverse outcomes are cirrhosis and hepatocellular carcinoma, consequently may cause liver-related death. Here, this article focuses on viral hepatitis because viruses mainly including hepatitis B virus (HBV), and hepatitis C virus (HCV), which have already been proved to be the most common cause of hepatitis worldwide. Furthermore, about a million of patients die from chronic viral hepatitis, most of which occur indirectly from liver scarring or liver cancer [1]. Therefore, early diagnosis of chronic viral hepatitis not only plays a significant role in hepatitis treatment and prevention but also allows for inhibiting disease progression, and decreasing transmission to others to a large extent [1,2].

Nowadays, the laboratory diagnostic tests for hepatitis are mainly based on blood tests, imaging, and liver biopsy [3]. Blood tests include antigens, antibodies, liver-associated enzymes, nucleic acid testing (e.g. hepatitis virus DNA/RNA). Furthermore, hepatic ultrasound, computed tomography, and magnetic resonance imaging are the procedures which can identify steatosis of the liver tissue and nodularity of the liver surface suggestive of cirrhosis. Liver biopsy is still the gold standard for assessing the precise extent and pattern of inflammation and fibrosis of the liver. However, some of these diagnostic approaches are invasive while some fail in early detection of disease due to the limited sensitivity which can only be used in advanced cases [4]. Since the delayed diagnosis may result in poor prognosis, it is necessary to find minimally invasive and cost-effective biomarkers to expand the diagnosis range for liver diseases. Accumulating evidence has witnessed the potential role of circulating miRNAs, as a part of “liquid biopsy”, in the diagnostic value for viral hepatitis [5]. miRNAs, a family of highly conserved single-stranded RNA molecules (19–22 nucleotides), have been proved to participated in multiple biological processes mainly including cell cycle, cell proliferation, differentiation, and apoptosis through binding to the complimentary 3′UTR of their target mRNAs and degrading the mRNAs [6,7]. Several research groups have demonstrated that circulating miRNAs, deriving from intracellular miRNAs and secreted out of the cell via exosomes and microvesicles during the process of cell death, can be stably detected in the biofluid like serum, plasma, urine, and cerebrospinal fluid (CSF) from patients making them relatively non-invasive biomarker for infectious diseases [8–10]. As a major miRNA in liver, miR-122 accounts for approximately 70% of the total liver miRNAs and has been widely reported to suffer from dysregulation in HCV and HBV infection. Besides, the level of miR-122 has been shown to correlate with the severity and stage of infection and helps to evaluate the treatment response [11–13]. In order to verify the hypothesis and assess the diagnostic value of miR-122 in chronic viral hepatitis, we systematically reviewed the literature and conducted this meta-analysis.

## Materials and methods

### Search strategy

In order to retrieve all the articles analyzing the diagnostic value of miR-122 in patients with HBV and/or HCV chronic viral hepatitis, a comprehensive literature search (updated to January 30, 2019) in PubMed, Cochrane library, EMBASE, and CNKI Wanfang and CQVIP databases was performed without language restrictions. The medical subject heading (MeSH) terms (“microRNA-122” or “miRNA-122” or “miR-122” or “hsa-miR-122”) and (“diagnostic value” or “diagnoses” or “receiver operating characteristics curve” or “ROC curve” or “sensitivity and specificity”) and (“HCV” or “Chronic hepatitis C” or “CHC” or “HBV” or “Chronic hepatitis B” or “CHB”) were used to identify all the relevant articles. Besides, we examined the reference lists of review articles and selected papers manually to identify whether there are any other eligible articles.

### Inclusion and exclusion criteria

Studies were considered eligible for inclusion in this meta-analysis had to fulfill the following criteria: (1) evaluate the diagnostic value of miR-122 in patients with HBV and/or HCV-associated chronic viral hepatitis; (2) the patients with HBV-associated chronic viral hepatitis should be positive for HBV surface antigen, positive for HBV DNA, while the diagnosis of HCV-associated chronic viral hepatitis was based on the detection of anti-HCV antibodies and consistent detection of HCV RNA, for at least 6 months; (3) each study involved both experimental and control groups; (4) the miR-122 expression was measured in serum or plasma samples; (5) articles provided sufficient data. Exclusion criteria were as follows: (1) studies with duplicate data; (2) meta-analysis, letters, reviews, case reports; (3) studies without sufficient data or with 20 patients or less.

### Data extraction and quality assessment

Two investigators (XHZH and SHQF) are responsible for assessing all the publications. Any disagreements were resolved through discussion with a third reviewer (MW). The following information was extracted from eligible studies: first author’s name, year and country of the publication, characteristics of participants (ethnicity, total number of cases and controls, mean/median age), sample types, methods of miR-122 testing, and the data (true-positive (TP), false-positive (FP), false-negative (FN), and true-negative (TN), sensitivity, and specificity). In addition, the Quality Assessment of Diagnostic Accuracy Studies 2 (QUADAS-2) was performed to assess the methodological qualities of each included article.

### Statistical analysis

Stata 14.0 (STATA Corp, College Station, TX, U.S.A.) and Meta-DiSc version 1.4 software were used to perform all the statistical analysis and *P*-value less than 0.05 was considered statistically significant. We calculated the pooled sensitivity, specificity, positive likelihood ratio (PLR), negative likelihood ratio (NLR), diagnostic odds ratio (DOR), and corresponding 95% confidence intervals (CIs) for the miR-122 studied using the bivariate random-effect regression model [14]. In addition, we determined the sensitivity and specificity of the miRNAs in each study using a bivariate summary receiver operating characteristic (SROC) curve. We calculated the area under the curve (AUC) and the maximum point of intersection between sensitivity and specificity [15]. Moreover, subgroup and sensitivity analysis were carried out to explore potential sources of between-study heterogeneity. And publication bias was assessed by Deeks’ funnel plot asymmetry test.

## Results

### Selection process and characteristics of the eligible studies

**Figure 1:**
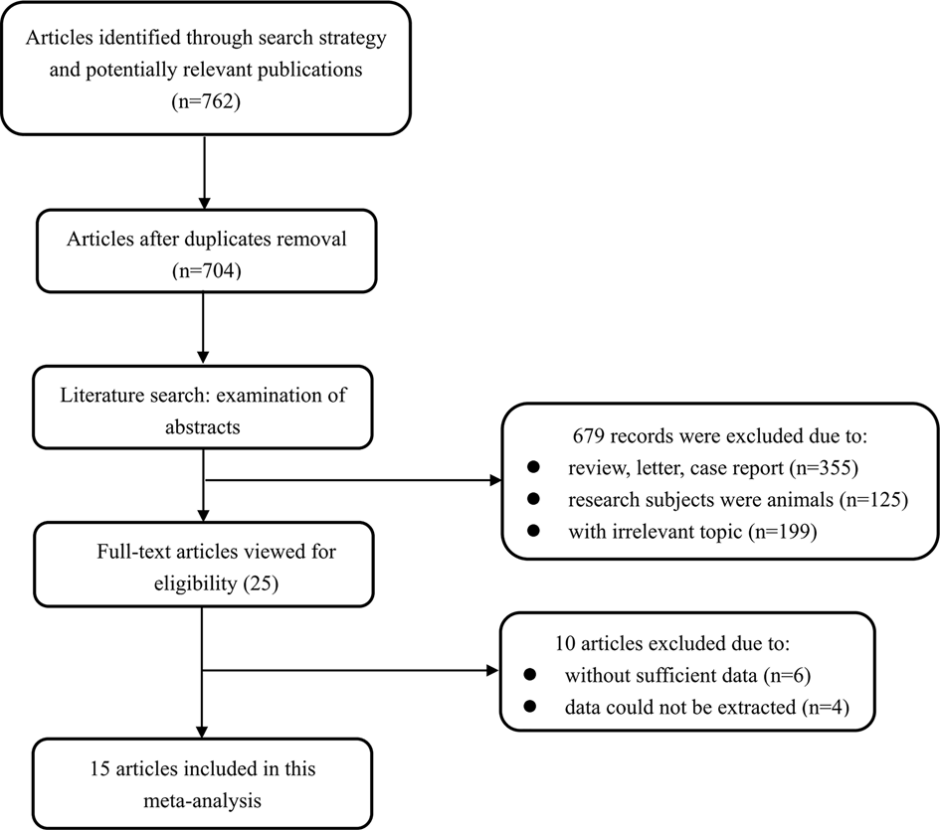
Flowchart of the articles selection process in this meta-analysis

As presented in Figure 1, a total of 762 articles were initially identified from the primary literature search strategy. 704 articles were left for screening after 65 duplicates were removed through Endnote X7 software. After reviewing their abstracts and titles, 679 articles were removed due to unfit literary forms, research subjects of animal model and irrelevant research topic. Subsequently, the full-texts of the remaining 25 articles were read to assess the eligibility and 10 articles were further excluded. As a result, 15 articles were included in the current meta-analysis [13,16–29].

**Figure 2:**
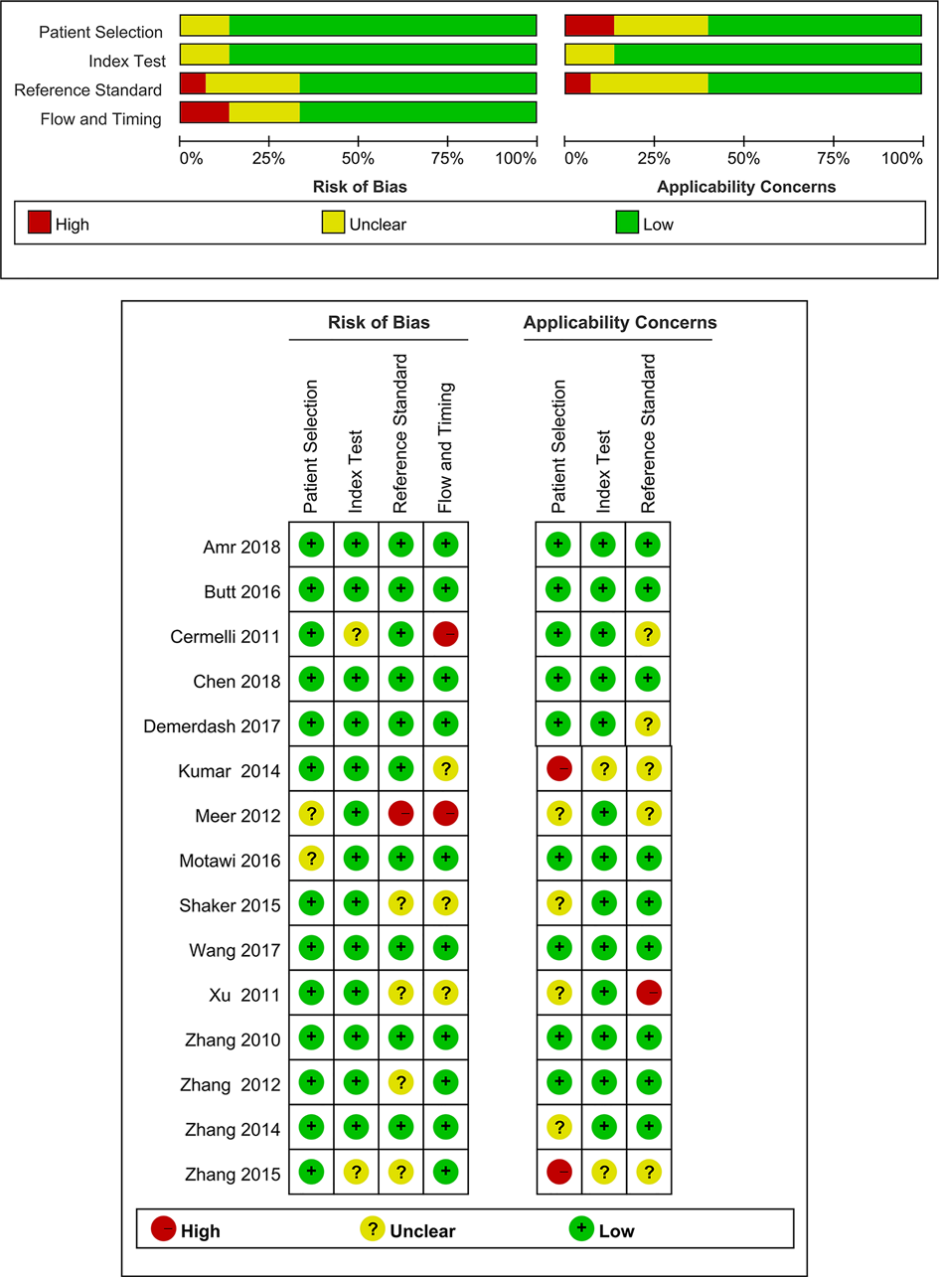
Overall methodological quality assessments of the included 15 articles based on QUADAS-2 tool

The main characteristics, along with the QUADAS-2 scores of the 15 included articles are summarized in Table 1, in an order by the publication year, ranging from 2010 to 2018. In all studies, quantitative real time polymerase chain reaction (qRT-PCR) assays were performed to detect the expression levels of miRNAs in either plasma (*n* = 4) or serum (*n* = 11). Seven articles of these selected studies were conducted in China, while the rest eight articles did research in foreign countries. As for the virus types, six articles focus on HBV-associated chronic viral hepatitis, and nine articles focus on HCV-associated chronic viral hepatitis. In addition, according to the 14-item QUADAS assessment tool, the quality assessments for each included study are presented in Figure 2.

**Table 1:** The summary characteristics and quality assessment of diagnostic clinical trials included in this meta-analysis

Included studies	Country	Detection method	Internal reference	Source of the virus	Sample size		Mean age (year)		Sensitivity	Specificity	Specimen	QUADAS-2
					Case	Control	Case age	Control age				
Zhang, 2010	China	SYBR PCR	U6 snRNA	HBV	83	40	40.2 ± 13.1	39.1 ± 13.4	98%	93%	Plasma	9
Xu, 2011	China	SYBR PCR	U6 snRNA	HBV	48	89	NA	NA	80%	96%	Serum	6
Cermelli, 2011	Egypt	SYBR PCR	miR-238	HCV	18	19	NA	NA	94%	83%	Serum	7
Meer, 2012	Netherlands	Taqman PCR	NA	HCV	102	25	48.65 ± 10.34	35.3 ± 11.5	95%	92%	Serum	5
Zhang, 2012	China	SYBR PCR	NA	HBV	24	24	37.6 ± 9.0	35.6 ± 10.2	88%	100%	Serum	6
Kumar, 2014	India	Taqman PCR	U6 snRNA	HCV	25	25	38.08 ± 10.81	32.53 ± 9.63	92%	84%	Serum	6
Zhang, 2014	China	SYBR PCR	U6 snRNA	HBV (active)	112	22	NA	NA	86%	63%	Plasma	7
	China	SYBR PCR	U6 snRNA	HBV (indolent)	19	22	NA	NA	84%	37%	Plasma	
Zhang, 2015	China	SYBR PCR	U6 snRNA	HCV	39	29	49.0 ± 14.3	45.0 ± 16.1	92%	79%	Serum	6
Shaker, 2015	Egypt	SYBR PCR	SNORD68	HCV	30	55	60.27 ± 8.2	55.88 ± 15.91	90%	100%	Serum	7
Motawi, 2016	Egypt	SYBR PCR	SNORD68	HCV	40	30	42.95 ± 11.21	49.9 ± 14.9	93%	100%	Serum	7
Butt, 2016	Egypt	SYBR PCR	U6 snRNA	HCV (abnormal ALT)	80	60	32.7 ± 9.9	39.2 ± 12.9	87%	97%	Serum	8
	Egypt	SYBR PCR	U6 snRNA	HCV (normal ALT)	43	60	32.7 ± 9.9	39.2 ± 12.9	65%	93%	Serum	
Demerdash, 2017	Egypt	SYBR PCR	SNORD68	HCV	60	60	35.1 ± 6.7	33.9 ± 8.64	80%	88%	Plasma	7
Wang, 2017	China	Taqman PCR	cel-miR-39	HBV (occult)	119	117	42.30 ± 13.60	45.58 ± 13.08	79%	55%	Serum	9
	China	Taqman PCR	cel-miR-39	HBV (active)	115	117	42.30 ± 13.60	44.40 ± 13.10	86%	83%	Serum	
Amr, 2018	Egypt	SYBR PCR	SNORD68	HCV	50	20	41.5	41.7	72%	85%	Serum	8
Chen, 2018	China	Taqman PCR	Hsa-miR-25-3p	HBV	30	30	42.7 ± 10.3	37.6 ± 12.8	80%	83%	Plasma	8

NA, not available.

### Diagnostic accuracy of miR-122 in chronic viral hepatitis

**Figure 3:**
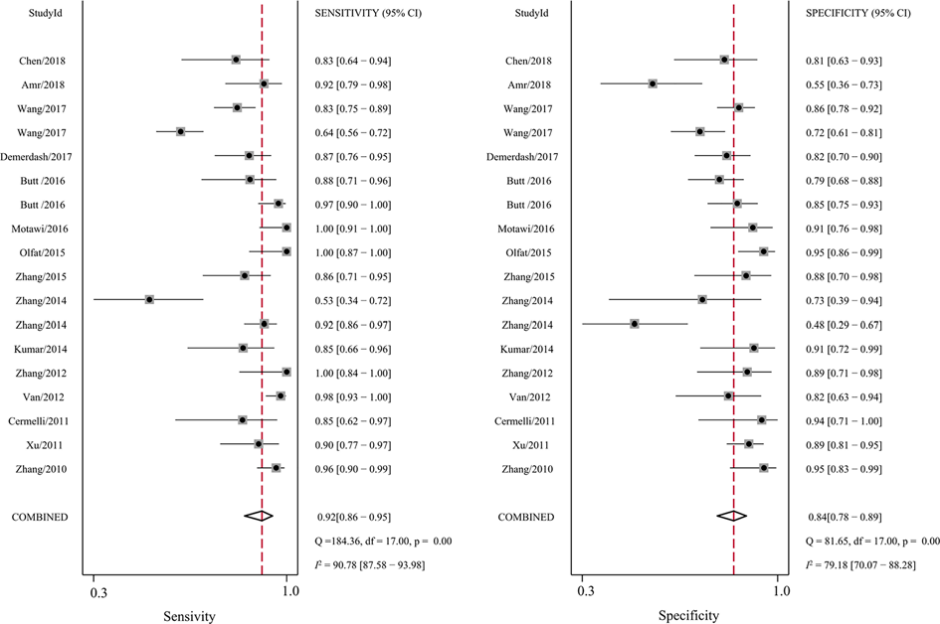
Forest plots of summary sensitivities and specificity of circulating miR-122 in the diagnosis of HBV- and HCV-associated chronic viral hepatitis

**Figure 4:**
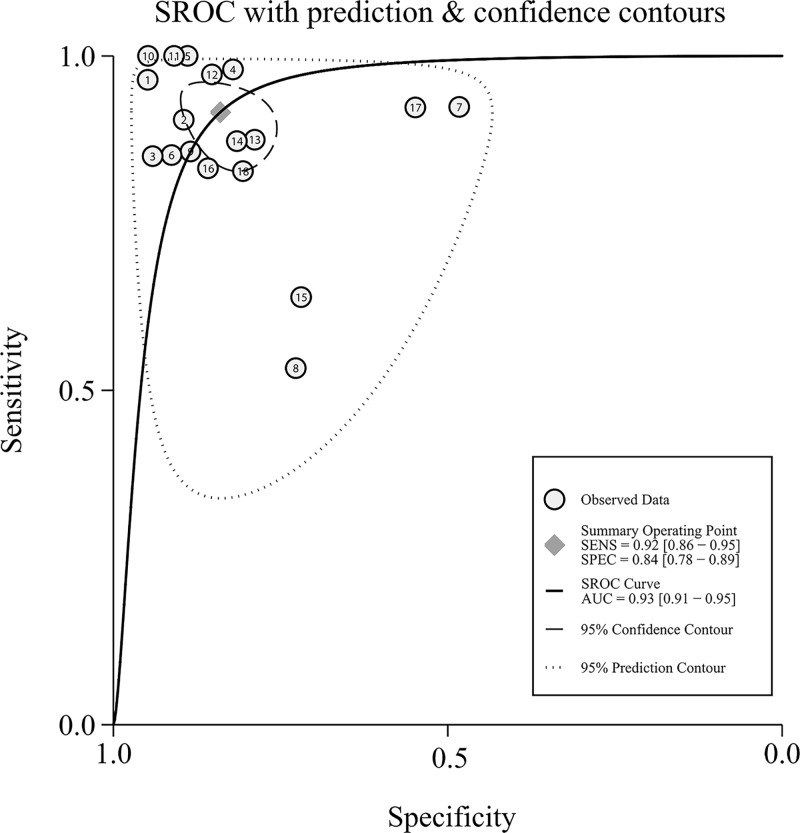
Summary ROC curves for miR-122 in the diagnosis of HBV- and HCV-associated chronic viral hepatitis

First, significant heterogeneity was found in our meta-analysis, as demonstrated by the results (*I*^2^ = 90.78% for sensitivity and *I*^2^ = 79.18% for specificity, respectively). Thus, the random-effect model was selected for the next calculation. Forest plots of the sensitivity and specificity results are shown in Figure 3. Overall, as shown in Table 2, the pooled sensitivity was 0.92 (95%CI: 0.86–0.95), specificity was 0.84 (95% CI: 0.78–0.89), PLR was 5.7 (95%CI: 4.7–8.1), NLR was 0.10 (95%CI: 0.06–0.18), DOR was 57 (95%CI: 25–129), and AUC was 0.93 (95%CI: 0.91–0.95). The SROC curve for the overall results is shown in Figure 4. The above results both suggest that miR-122 can be served as an adjuvant tool for the diagnosis of HBV- and/or HCV-associated chronic viral hepatitis.

**Table 2:** Summary diagnostic accuracy of circulating miR-122 for HBV and/or HCV

Analysis	Sensitivity (95% CI)	Specificity (95% CI)	PLR (95% CI)	NLR (95% CI)	DOR (95% CI)	AUC (95% CI)
*Virus types*						
HBV	0.87 (0.75–0.94)	0.81 (0.75–0.87)	4.7 (3.3–6.7)	0.16 (0.07–0.33)	30 (11–79)	0.88 (0.85–0.91)
HCV	0.94 (0.89–0.97)	0.85 (0.78–0.90)	6.6 (4.4–10.0)	0.07 (0.04–0.14)	89 (36–217)	0.95 (0.93–0.97)
*Sample types*						
Serum	0.93 (0.86–0.97)	0.86 (0.80–0.90)	6.4 (4.5–9.2)	0.08 (0.04–0.17)	79 (30–207)	0.94 (0.91–0.96)
Plasma	0.87 (0.72–0.95)	0.79 (0.61–0.90)	4.1 (2.0–8.5)	0.16 (0.07–0.40)	25 (6–109)	0.90 (0.87–0.92)
*Ethnicity*						
Chinese	0.87 (0.76–0.93)	0.83 (0.73–0.89)	5.1 (3.0–8.5)	0.16 (0.08–0.31)	32 (11–95)	0.91 (0.88–0.93)
Non-Chinese	0.95 (0.89–0.97)	0.85 (0.77–0.91)	6.6 (4.4–10.0)	0.06 (0.03–0.13)	100 (36–279)	0.96 (0.94–0.97)
Overall	0.92 (0.86–0.95)	0.84 (0.78–0.89)	5.7 (4.7–8.1)	0.10 (0.06–0.18)	57 (25–129)	0.93 (0.91–0.95)
Outliers excluded	0.93 (0.87–0.95)	0.86 (0.82–0.89)	6.6 (4.9–8.9)	0.09 (0.05–0.19)	66 (27–160)	0.94 (0.90–0.96)

### Threshold effect and subgroup analysis

Since threshold effect has been reported to be major cause of between-study heterogeneity and occurs when differences in sensitivities and specificities arise; this effect can be assessed with the spearman correlation coefficient [30]. And a value of −0.298 (*P*=0.229; *P*>0.05) indicated the absence of the threshold effect in our study. Afterwards, subgroup analyses based on ethnicity, sample types and virus types were also performed. The pooled sensitivity, specificity, PLR, NLR, DOR, and AUC for each subgroup are listed in Table 2. As for virus types, the HCV-associated chronic viral hepatitis group showed a higher accuracy than HBV-associated chronic viral hepatitis group with sensitivity of 0.94 versus 0.87, specificity of 0.85 versus 0.81, PLR of 6.6 versus 4.7, NLR of 0.07 versus 0.16, DOR of 89 versus 30 and AUC of 0.95 versus 0.88, respectively. Furthermore, a comparison of miR-122 expression profile in serum and plasma showed that the sensitivity (0.93 versus 0.87), specificity (0.86 versus 0.79), and AUC (0.94 versus 0.90) were higher in serum-based test than in plasma, providing additional evidence for the use of serum as a better matrix for diagnostic profiling of miR-122 in patients with HBV- and/or HCV-associated chronic viral hepatitis. Afterwards, the analysis based on ethnicity demonstrated the non-Chinese populations yield a better diagnosis accuracy than their Chinese counterparts. Specifically, for the non-Chinese population group, the pooled sensitivity, specificity, PLR, NLR, DOR, and AUC were 0.95, 0.85, 6.6, 0.06, 100, and 0.96, while the results for the Chinese population group were 0.97, 0.83, 5.1, 0.16, 32, and 0.91, respectively.

### Sensitivity analysis and publication bias

**Figure 5:**
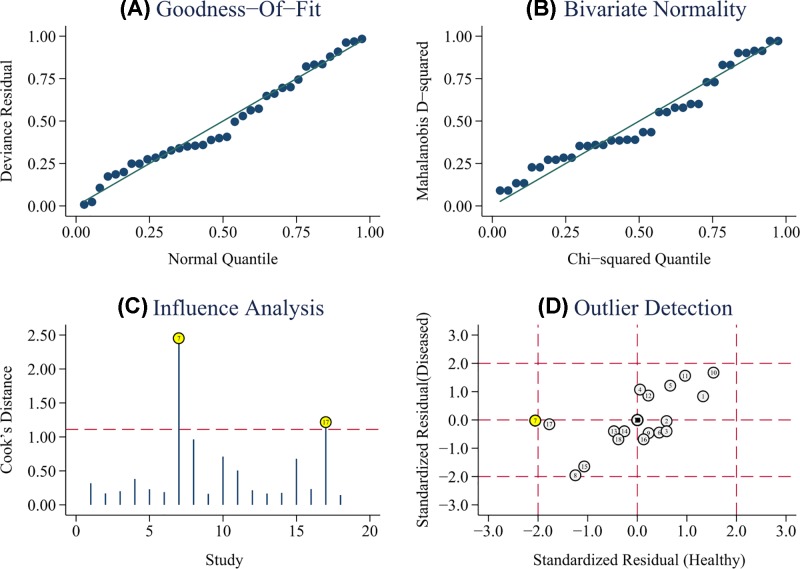
Sensitivity analysis: graphical depiction of goodness of fit and bivariate normality analysis (**A,B**), influence and outlier detection analysis (**C,D**), respectively

**Figure 6:**
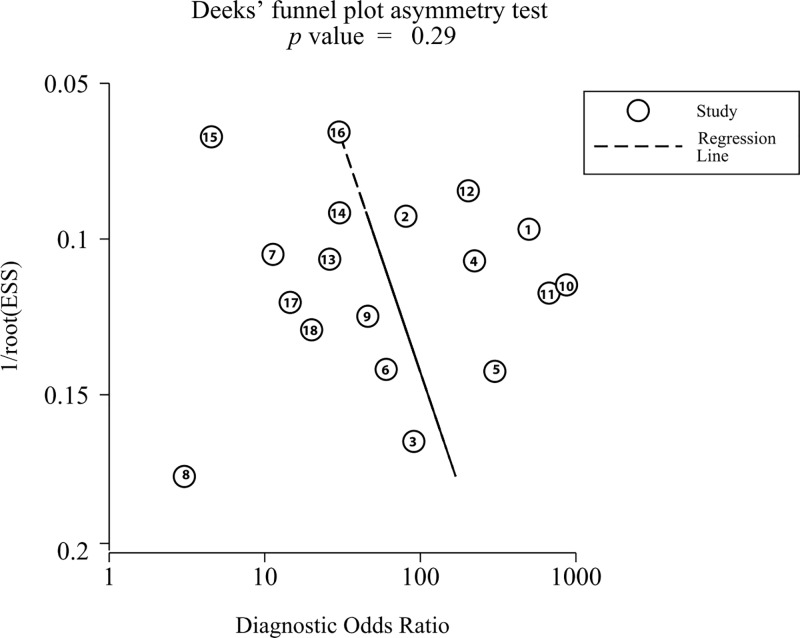
Deeks’ funnel plot asymmetry test for the assessment of potential publication bias

As shown in Figure 5A,B, the results of goodness of fit and bivariate normality analysis suggested that the random-effect model was suitable for subsequent calculation of the pooled estimates. Afterwards, influence analysis and outlier detection (Figure 5C,D) identified two outlier researches. After excluding these outliers, sensitivity increased from 0.92 to 0.93, specificity increased from 0.84 to 0.86, PLR increased from 5.7 to 6.6, NLR decreased from 0.10 to 0.09, DOR increased from 57 to 66, and AUC increased from 0.93 to 0.94. In addition, heterogeneity increased from 90.78% to 91.19% for sensitivity and from 79.18% to 62.02% for specificity. Furthermore, Deeks’ funnel plot asymmetry test was carried out to explore the potential publication bias of the included studies. As demonstrated in Figure 6, an obtained *P* value of 0.29 indicated the absence of publication bias in this meta-analysis.

## Discussion

Though significant progress has been occurred in diagnostic techniques over the years, the accurate and convenient diagnosis of HBV- and/or HCV-associated chronic viral hepatitis remains a clinical challenge. Subsequently, the application of miRNAs, which can control gene expression in multiple biological processes including growth, cell proliferation, differentiation, apoptosis, and carcinogenesis through RNA interference (RNAi) [31,32], has gained much attention. As a part of miRNAs, miR-122 has already been demonstrated to be the most frequent miRNA in adult human liver accounting up to 70% of the total hepatic miRNAs, and a central player in liver development, differentiation, and homeostasis as well as in metabolic functions [33]. There is a great deal of researches into the use of miR-122 as a biomarker for HBV and HCV. In the aspect of HBV regulation, Chen et al. [34] have demonstrated that miR­122 can bind to the highly conserved region of a bicistronic mRNA called HBV pregenomic RNA, which reported to encode the HBV polymerase and core protein, thereby ultimately leading to inhibition of HBV gene expression and replication. Furthermore, it has also been confirmed that increase in miR­122 can attenuated the replication of HBV by regulating cyclin G(1) -modulated P53 activity [35]. As for the aspect of HCV regulation, liver-specific miR­122 can stabilize HCV viral RNA through a process involving protecting highly conserved 5′ untranslated region the HCV genome from degradation by the host exonuclease, Xrn­1 or from host innate immune responses [36–39]. A phase 2a study has shown that miravirsen, an antisense oligonucleotide, exhibited remarkable prolonged dose-dependent reductions in HCV RNA levels in patients with chronic HCV genotype 1 infection [40]. All the above researches both suggest that miR­122 is a significant regulator of HBV and HCV replication by either directly affecting viral RNA or modulating host gene expression.

However, due to the different study designs and study subjects, some studies dispute its diagnostic efficacy [41]. Thus, the present study was carried out to summarize the results of individual studies. As the present results show, circulating miR-122 achieved a pooled sensitivity of 0.92, specificity of 0.84, and AUC of 0.93, indicating an overall moderate test performance for the diagnosis of HBV- and HCV-associated chronic viral hepatitis. Furthermore, the PLR value of 5.7 suggested that HBV- and HCV-associated chronic viral hepatitis patients had an almost six-fold higher chance of being miR-122 test positive than other individuals without the disease, and a NLR value of 0.1 implied that in a negative result from the miR-122 test, only 10% is likely to be false-negative. Obviously, the DOR value, which combines the strengths of both sensitivity and specificity, was 57 in our meta-analysis, indicating a high level of discriminating accuracy for clinical practice [42].

Furthermore, to explore the potential sources of heterogeneity, subgroup analysis based on virus types, sample types and ethnicity were subsequently performed. The results of subgroup analysis based on virus types suggest that miRNA-122 yielded an overall higher diagnostic accuracy in HCV-associated chronic viral hepatitis patients with a sensitivity of 0.94, specificity of 0.85, PLR of 6.6, NLR of 0.07, DOR of 89, and AUC of 0.95. Besides, with regard to sample types, miRNA expression profiles have been reported to be considerably affected by the coagulation process in the blood [43]. In our study, serum turned out to be a better matrix for diagnostic profiling of miR-122 in HBV- and HCV-associated chronic viral hepatitis than plasma: the pooled sensitivity was 0.93 versus 0.87, specificity was 0.86 versus 0.79, PLR was 6.4 versus 4.1, NLR was 0.08 versus 0.16, DOR was 79 versus 25, and AUC was 0.94 versus 0.90. However, as only four studies were included in the plasma specimen group, thus large-scale prospective researches are still needed to consolidate the results. In the subgroup analysis based on ethnicity, we found compared with Chinese populations, miR-122 assay may be more accurate in non-Chinese populations, with the DOR value hiked from 32 to 100, and AUC increased from 0.91 to 0.96.

Admittedly, heterogeneity still exists when interpreting the results of any meta-analysis. In our study, heterogeneity does not come from the threshold effect. However, in the sensitivity analysis, after excluding the outlier, the overall pooled sensitivity, sensitivity, PLR, DOR, and AUC all increased while NLR decreased suggesting that the outlier is probably a source of heterogeneity. In addition, as the *P* value of *I*^2^ for the overall study altered inconspicuously, substantial heterogeneity from non-threshold effect still exists among studies to some extent. Although we have made every effort to avoid bias during the process of our study, there were still some limitations in this meta-analysis. In the first place, several valuable studies may be missed in spite of the comprehensive search strategy during our literature search. What’s more, diagnostic performance maybe affected as the majority of healthy people were randomly selected as controls and were not blind designed.

## Conclusions

Taken together, for the first time, our meta-analysis focuses on the diagnostic performance of circulating miRNA-122 in HBV- and/or HCV-associated chronic viral hepatitis detection, and it is concluded that circulating miRNA-122 has a relatively high diagnostic value for chronic viral hepatitis detection, especially in patients of HCV-associated chronic viral hepatitis. However, in the future, well-designed, large-scale and accurate researches are still needed to consolidate the results of this meta-analysis in clinical practice. The ultimate purpose is to combine clinical variables with those available in public databases to open avenues for prospective trials of a non-invasive diagnostic test for enhancing the early detection of patients with chronic viral hepatitis.

## Abbreviations

AUC: area under the curve
CI: confidence interval
DOR: diagnostic odds ratio
HBV: hepatitis B virus
HCV: hepatitis C virus
NLR: negative likelihood ratio
PLR: positive likelihood ratio
QUADAS: Quality Assessment of Diagnostic Accuracy Studies
SROC: summary receiver operating characteristic
